# CD133-positive hepatocellular carcinoma in an area endemic for hepatitis B virus infection

**DOI:** 10.1186/1471-2407-9-324

**Published:** 2009-09-11

**Authors:** Chau-Ting Yeh, Chia-Jung Kuo, Ming-Wei Lai, Tse-Ching Chen, Chun-Yen Lin, Ta-Sen Yeh, Wei-Chen Lee

**Affiliations:** 1 Liver Research Unit, Department of Hepato-Gastroenterology, Chang Gung Memorial Hospital, Taipei, Taiwan, Republic of China; 2 Molecular Medicine Research Center, Chang Gung University, Taoyuan, Taiwan, Republic of China; 3 Department of Pediatric Gastroenterology, Chang Gung Children Hospital, Taoyuan, Taiwan, Republic of China; 4 Department of Pathology, Chang Gung Memorial Hospital, Taipei, Taiwan, Republic of China; 5 Division of General Surgery, Department of Surgery, Chang Gung Memorial Hospital, Taipei, Taiwan, Republic of China

## Abstract

**Background:**

CD133 was detected in several types of cancers including hepatocellular carcinoma (HCC), which raised the possibility of stem cell origin in a subset of cancers. However, reappearance of embryonic markers in de-differentiated malignant cells was commonly observed. It remained to be elucidated whether CD133-positive HCCs were indeed of stem cell origin or they were just a group of poorly differentiated cells acquiring an embryonic marker. The aim of this study was to investigate the significance of CD133 expression in HCC in an area endemic for hepatitis B virus (HBV) infection to gain insights on this issue.

**Methods:**

154 HCC patients receiving total removal of HCCs were included. 104 of them (67.5%) were positive for HBV infection. The cancerous and adjacent non-cancerous liver tissues were subjected for Western blot and immunohistochemistry analysis for CD133 expression. The data were correlated with clinical parameters, patient survivals, and p53 expression.

**Results:**

Of 154 patients, 24 (15.6%) had CD133 expression in HCC. Univariate and multivariate logistic regression analysis revealed that CD133 expression was negatively correlated with the presence of hepatitis B surface antigen (HBsAg). The unadjusted and adjusted odds ratios were 0.337 (95%CI 0.126 - 0.890) and 0.084 (95%CI 0.010 - 0.707), respectively. On the other hand, p53 expression was positively associated with the presence of HBsAg in univariate analysis. The unadjusted odds ratio was 4.203 (95%CI 1.110 - 18.673). Survival analysis indicated that both CD133 and p53 expression in HCC predicted poor disease-free survival (P = 0.009 and 0.001, respectively), whereas only CD133 expression predicted poor overall survival (P = 0.001). Cox proportional hazard model showed that p53 and CD133 expression were two independent predictors for disease-free survival. The hazard ratios were 1.697 (95% CI 1.318 - 2.185) and 2.559 (95% CI 1.519 - 4.313), respectively (P < 0.001 for both).

**Conclusion:**

In area where HBV infection accounts for the major attributive risk of HCC, CD133 expression in HCC was negatively associated with the presence of HBsAg, implicating a non-viral origin of CD133-positive HCC. Additionally, CD133 expression predicted poor disease-free survival independently of p53 expression, arguing for two distinguishable hepatocarcinogenesis pathways.

## Background

Hepatocellular carcinoma (HCC) is the fifth most commonly diagnosed solid cancer as well as the third most common cause of cancer-related death in the world [1]. The etiology of HCC is multifactorial, including chronic hepatitis B virus (HBV) infection, chronic hepatitis C virus (HCV) infection, alcoholic liver disease, and others [2,3]. In areas where chronic hepatitis B is highly prevalent, such as Southeast Asia and sub-Saharan Africa, correspondingly higher incidence of HCC is found [4]. In contrast, in Western countries, where chronic hepatitis C and alcoholic liver disease account for the major attributable risk, lower prevalence of HCC is observed. Over the past two decades, the incidence of HCC is rising in Western world, possibly due to increased incidence of HCV infection [5]. Other risk factors include old age, male gender, underlying chronic liver diseases, and most importantly, liver cirrhosis [6]. Aflatoxin exposure has also been linked to development of HCC in some areas [7-10]. A single mutation in codon 249 of p53 is frequently seen in this subgroup [9,10]. Because of the multifactorial etiology and heterogeneous nature of the diseases, the key molecular pathways leading to hepatocarcinogenesis remained illusive. With the help of advanced technologies in genomic medicine, it is discovered that hepatocarcinogenesis involved not only multiple steps of molecular events but also heterogeneous cellular pathways [11-13]. At this stage, it is pivotal to identify major subpopulations of HCC, of which the oncogenic pathways are better defined so that a specific targeted therapeutic modality can be devised.

CD133 is a human homologue of mouse Prominin-1, a five-transmembrane cell surface glycoprotein [14-16]. It is expressed in a subpopulation of the CD34^+ ^hematopoietic stem cells derived from fetal liver or bone marrow [17,18]. CD133 has been detected in neuroepithelial cells, embryonic epithelial cells and adult immature epithelial cells [19-21]. On the other hand, CD133 is expressed in several types of tumor tissues, including acute myeloid leukemia, glioblastoma, ependymoma and prostate cancer [22-29]. It is hypothesized that cancer can be originated and maintained by a subset of cancer stem cells [30,31]. Recently, CD133 was detected in HCC cell lines as well as some human HCC tissues, suggesting a stem cell origin [32-34]. Additionally, CD133 expression in HCC is associated with poor prognosis [35,36]. In Southeast Asia, however, the most important etiology for HCC is HBV infection. It is unclear whether cancer stem cells play a role in HBV-related hepatocarcinogenesis. In the present study, we investigated the clinicopathological significance of CD133 expression in HCC in an area endemic for HBV infection. Additionally, we examined whether CD133 expression associated with p53 over-expression in HCC, another factor associated with poor prognosis [37,38].

## Methods

### Patients

This study was conducted under approval of the institutional review board, Chang Gung Medical Center. Under informed consent, 154 HCC patients receiving total removal of liver tumors from July 1998 to Aug 2005 in Chang Gung Medical Center, Taiwan, were included. All samples were frozen to -70°C immediately after surgical resection and stored in Tissue Bank, Chang Gung Medical Center until used. The following clinicopathological data were retrospectively reviewed: gender, age, HBV surface antigen (HBsAg), antibody against HCV (anti-HCV), liver cirrhosis status, alcoholism, Edmonson's histology grading, microvascular invasion, tumor capsule, number of tumor, microsatellites, largest tumor size, portal vein invasion, presence of ascites on surgery, alpha-fetoprotein (AFP), albumin, bilirubin, prothrombin time, creatinine, aspartate aminotransferase (AST), alanine aminotransferase (ALT), the date of surgical resection, the date of tumor recurrence or metastasis, and the date of last follow-up or HCC related death.

Preoperative diagnosis of HCC was made by one of the following methods: echo-guided liver biopsy, fine needle aspiration cytology, high AFP level (> 200 ng/mL) plus at least one dynamic imaging studies (dynamic computed tomography or magnetic resonance imaging), or two dynamic imaging studies plus angiography (if AFP < 200 ng/mL). Tumors were totally removed with a safety-margin of > 1 cm. Post-operative follow-up was performed by ultrasonography, chest X-ray, AFP, and blood biochemistry every 1 to 3 months in the first year and 3 to 6 months thereafter. Abnormal findings were verified by computed tomography or magnetic resonance imaging. Intrahepatic recurrence was verified by use of the aforementioned criteria. Extrahepatic recurrence was verified by biopsy, aspiration cytology, computed tomography or magnetic resonance imaging study dependent on the location of the lesions as well as the condition of the patients.

HBsAg was measured by a radioimmunoassay (Ausria-II, HBsAg-RIA, Abbott Laboratories, North Chicago, IL). Anti-HCV was measured by a third-generation enzyme immunoassay (HCV EIA III; Abbott Laboratories).

### Western blot analysis

Protein was extracted from the cancerous and adjacent non-cancerous tissues with a commercial kit (T-PER Tissue Protein Extraction Reagent, Thermo Fisher Scientific Inc., Rockford, IL). After addition of a cocktail protease inhibitor mix (Halt Protease Inhibitor Cocktail Kit, Thermo Fisher Scientific Inc., Rockford, IL), the samples were stored at -80°C. After sodium dodecyl sulfate polyacrylamide gel electrophoresis, immunoblot was carried out by use of a primary mouse anti-human CD133 antibody (Miltenyi Biotec GmbH, Bergisch Gladbach, Gemany) in a 1 to 300 dilution for detection of CD133, or a primary rabbit anti-human p53 antibody (Abcam, Cambridge, UK) in a 1 to 200 dilution for detection of p53. After incubation with a secondary anti-mouse or anti-rabbit antibody, HRP conjugated (Leinco Technologies Inc., St. Louis, MO), the membrane was developed in chemiluminescent detection reagents (ECL Plus, GE Healthcare, Piscataway, NJ) and the signal was quantified by densitometry. Subsequently, the same membrane was stripped and re-probed with a primary mouse anti-human actin monoclonal antibody (Abcam, Cambridge, UK) in a 1 to 5,000 dilution for detection of actin.

### Immunohistochemistry

Liver specimens were fixed in 10% formaldehyde and embedded in paraffin. Five-micrometer paraffin sections were mounted on poly-L-lysine-coated slides for immunohistochemistry. After de-paraffinization in xylene, the sections were rehydrated through graded ethanol. Hepatocyte expression of CD133 was assessed by the avidin-biotin immunoperoxidase method. The sections were incubated in PBS containing 5% of hydrogen peroxide for 15 minutes and were subsequently washed twice (5 minutes each) in PBS containing 0.025% Triton X-100 (Sigma Chemical Co., St. Louis, MO). The tissue sections were then incubated with 3% of bovine serum albumin and 10% of normal goat serum for 30 minutes, followed by a 1:500 dilution of the same mouse antibody against human CD133 at 37°C for 1 hour. After washed with phosphate-buffered saline (PBS; 0.1 M, pH 7.4), the sections were subsequently incubated with biotin-conjugated goat anti-mouse immunoglobulins (Jackson Immunoresearch Lab., West Grove, PA) at a 1:400 dilution for 40 minutes. After rinsed with PBS, sections were treated with avidin-biotin complex (Vectastain Elite ABC Kit, Vector Labs, CA) for 30 minutes and then incubated in a diaminobenzidine solution (DAB, Vector Labs, CA) for 1 minute. Nuclear counterstaining was performed with hematoxylin.

### Real-time RT-PCR for CD133 mRNA quantification

Total RNA was isolated from both cancerous and non-cancerous liver tissues using TRI reagent (Molecular Research Center, Inc., Cincinnati, OH) according to the manufacturer's instructions. To eliminate contaminated DNA, the extracted RNA was dissolved in diethylpyrocarbonate-treated water containing 10 mmol/l of MgCl_2 _and incubated with 100 μg/ml of RNase-free DNase I for 30 min at 37°C. EDTA was added to a final concentration of 30 mmol/l, and the mixture was heated at 95°C for 5 min to stop the reaction. Reverse transcription was performed by use of random primers. The primers for PCR detection of CD133 cDNA were P1, 5'-TTTCAAGGACTTGCGAACTCTCTTGA-3' (nt. 776-801, sense) and P2, 5'-GAACAGGGATGATGTTGGGTCTCA (nt. 942-919, antisense). A fragment of amplified cDNA was inserted into the pRC2.1-TOPO vector (Invitrogen, Carlsbad, CA) and verified by DNA sequencing (CEQ 2000, Beckman Instruments Inc., Fullerton, CA). As a control, a fragment of β-actin cDNA flanked by PA1, 5'-CACCAACTGGGACGACATGG-3' (nt. 301-320, sense), and PA2, 5'-AGGATCTTCATGAGGTAGTC-3' (nt. 651-532, antisense), was amplified by RT-PCR and cloned into pRC2.1-TOPO.

The method of real-time PCR was described previously [39]. Briefly, CD133 RNA and β-actin RNA fragments were generated by in vitro transcription using the MEGAscript T7 kit (Ambion Inc., Austin, TA). The RNA product was thus dilated serially (10-fold) to generate a set of standards containing 10^6 ^to 10^1 ^copies. Real-time PCR was subsequently performed in LightCycler (Roche Diagnostics Corporation, IN). The copy numbers of these two RNA species derived from both the cancerous and non-cancerous tissues had to fall into the standard curves, otherwise both were diluted 10- and 100-fold in parallel and the assays were repeated. All data were obtained from two independent measurements.

### Statistical analysis

Correlation between the CD133 or p53 expression and clinicopathological parameters was conducted by use of Fisher's exact test or Chi-square test (for dichotomy data) and student's T test or linear regression analysis (for parametric data). Adjusted odds ratios (OR) were calculated using multivariate logistic regression analysis. Overall survival was calculated from the date of diagnosis to the date of death or last follow-up. Disease-free survival was measured from the date of diagnosis to the date of recurrence, metastasis, death or last follow-up. The Kaplan-Meier method was used to estimate the survival probability, and the log-rank test was used to compare the survival curves between groups. Independent predictive factors affecting survival were analyzed by the Cox multivariate proportional hazards model. P < 0.05 was considered statistically significant. Statistical analysis was conducted by use of SPSS (version 13.0).

## Results

### Western blot and immunohistochemistry analysis of CD133 expression in HCC

One hundred and fifty four patients, 40 females and 114 males, were included in this study. The basic clinical data were listed in Table 1. In females, HBsAg and anti-HCV were detected in about half of them (52.5% and 45.0%, respectively). In contrast, in males, HBsAg was detected in a higher proportion of patients (72.8%), whereas anti-HCV was detected in a lower proportion of patients (21.1%). A significantly higher proportion of males had documented alcoholic history. Of 154 patients, 14 (9.1%) had no known etiology for HCC. Interestingly, 8 of these 14 patients (57.1%) had diabetes. All patients positive for HBsAg were chronic carrier and the infection occurred before 30 years of age. All patients were negative for serum HBV e antigen (HBeAg). None of these patients had received antiviral therapy prior to the diagnosis of HCC.

**Table 1:** Basic clinical characterization of patients included

Clinicopathological parameters	Gender		P
		
	Female (n = 40)	Male (n = 114)	
Age (years)	57.5 ± 13.2	55.8 ± 15.5	0.545
Etiology			
HBsAg (positive)	21 (52.5%)	83 (72.8%)	0.030
Anti-HCV (positive)	18 (45.0%)	24 (21.1%)	0.007
Alcoholism (yes)	4 (10.0%)	39 (34.2%)	0.004
Unknown cause^a^	5 (12.5%)	9 (7.9%)	0.359
Cirrhosis (yes)	20 (50.0%)	53 (46.5%)	0.717
Tumor number			0.491^b^
1	28	72	
2	6	18	
3	5	17	
4	1	6	
5	0	1	
Tumor size (Diameter, cm)	7.0 ± 4.8	6.8 ± 4.7	0.826
Ascites (yes)	1 (2.5%)	13 (11.4%)	0.116

^a^Diabetes was found in 3 females and 5 males, respectively

^b^Comparison between patients with tumor number ≦ 2 and those with tumor number > 2.

**Figure 1:**
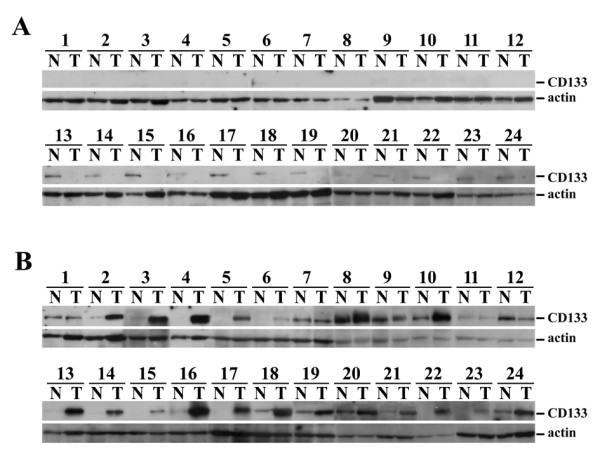
**Western blot analysis of CD133 in paired cancerous (T) and adjacent non-cancerous (N) liver tissues**. (A) CD133 was undetectable in the cancerous tissues. In some patients, a small amount of CD133 was detected in the non-cancerous parts (lower panel). (B) CD133 was detected in the cancerous liver tissues.

The cancerous and non-cancerous liver tissues were subjected for western blot analysis to detect CD133 expression (Figure 1). Three expression patterns were found. CD133 could be undetectable in both cancerous and non-cancerous tissues (Figure 1A, upper panel), a small amount of CD133 could be detected in non-cancerous tissues but not in cancerous tissues (Figure 1A, lower panel) or alternatively, a significant amount of CD133 was detected in cancerous tissues (Figure 1B). The latter pattern was observed in 24 patients.

**Figure 2:**
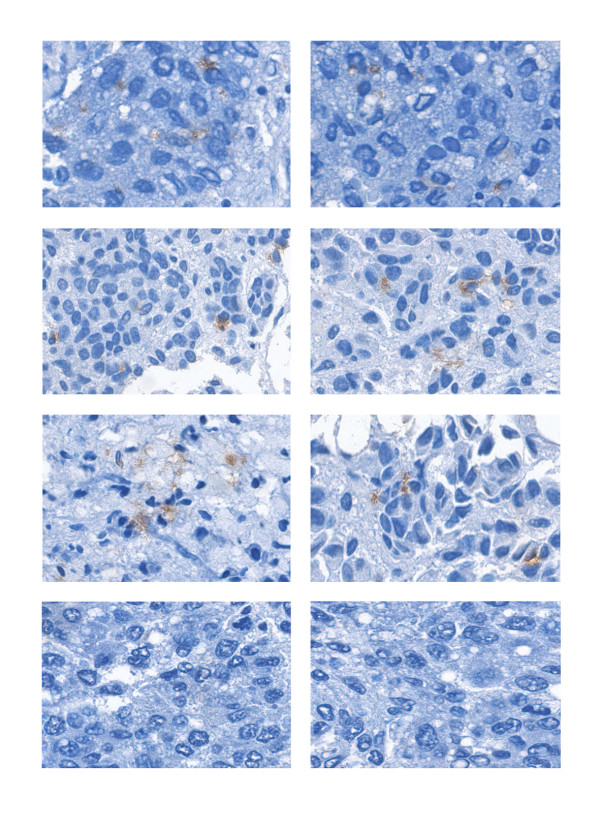
**Immunohistochemistry analysis of CD133 expression in HCCs**. CD133 was detected by avidin-biotin immunoperoxidase method. Nuclei were counterstained by hematoxylin. Original magnification, 400×. Two HCC samples negative for CD133 expression served as negative controls (bottom panel).

**Figure 3:**
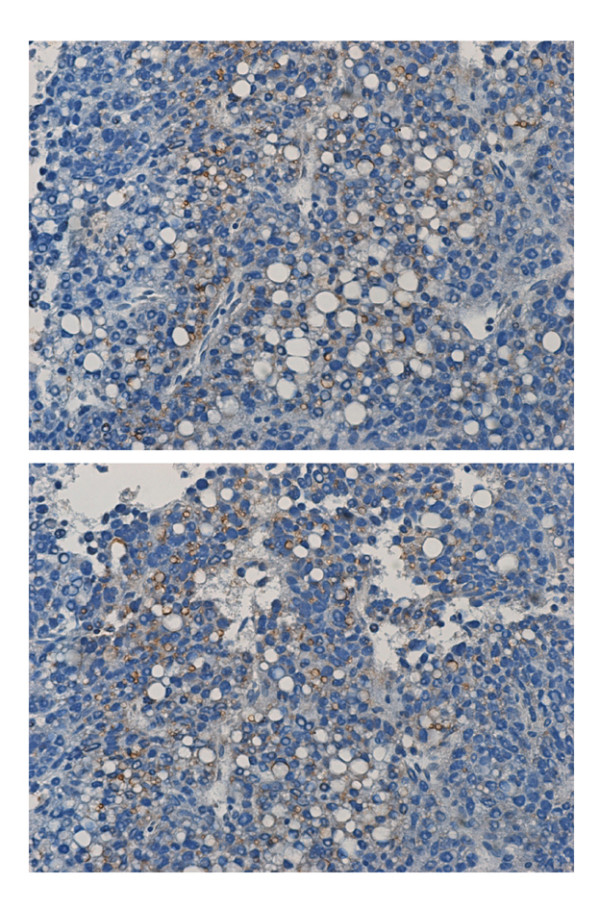
**Immunohistochemistry analysis of CD133 expression in HCC in patient-16**. Original magnification, 200×.

Immunohistochemistry analysis was subsequently performed. It was discovered that in the 24 patients positive for CD133 expression in HCC, CD133 was mostly accumulated as patches with granular appearance in the cytoplasm of a few scattered malignant hepatocytes (Figure 2). In a particular patient, who had the highest amount of CD133 expression in HCC (Figure 1B, patient-16), CD133 was detected in the margins of vesicles in a great proportion of cells (Figure 3). Additionally, marked fatty metamorphosis was observed. This patient was negative for serum HBsAg and anti-HCV.

### Real-time PCR assay for CD133 mRNA

For samples negative for CD133 expression by western blot analysis, the CD133 mRNA level was either undetectable or < 10^2 ^copies per 10^5 ^copies of β-actin mRNA. For non-cancerous tissues expressing a small amount of CD133 (Figure 1A, lower panel), the CD133 mRNA level was 10^2.3 ^± ^0.4 ^to 10^4.1 ^± ^0.6 ^copies per 10^5 ^copies of β-actin mRNA. For cancerous tissues positive for CD133 by western blot analysis (Figure 1B), the CD133 mRNA level was 10^2.5 ^± ^0.5 ^to 10^6.1 ^± ^0.9 ^copies per 10^5 ^copies of β-actin mRNA.

### CD133 expression in HCC is negatively associated with the presence of HBsAg

The clinicopathological data in patients with CD133 expression in HCC (n = 24) were compared with those without CD133 expression in HCC (n = 130) (Table 2). Among all factors compared, only the presence of HBsAg and tumor number > 2 were significant different in univariate analysis. The unadjusted OR (95% confidence interval [CI]) was 0.337 (0.126 - 0.890) and 3.114 (1.088 - 8.867), respectively (P = 0.025 and 0.032). However, in multivariate logistic regression analysis, only the presence of HBsAg remained significantly correlated with CD133 expression. The adjusted OR (95% CI) was 0.084 (0.010 - 0.707) (P = 0.023). Therefore, CD133 expression was negatively associated with the presence of HBsAg and the association was independent of other factors.

**Table 2:** Multivariate logistic regression analysis of clinical parameters associated with CD133 expression in HCC.

Clinicopathological parameters	CD133 expression in HCC		Unadjusted OR **(95% CI)**^**a**^	Adjusted OR **(95% CI)**^**b**^
			
	Positive (n = 24)	Negative (n = 130)		
Age (years)	53.6 ± 15.3	56.7 ± 14.9	0.983 (0.959 - 1.010)	0.997 (0.943 - 1.055)
Sex (male)	18 (75.0%)	96 (74.8%)	1.063 (0.358 - 3.282)	0.621 (0.109 - 3.542)
HBsAg (positive)	11 (45.8%)	93 (71.5%)	0.337 (0.126 - 0.890)^c^	0.084 (0.010 - 0.707)^d^
Anti-HCV (positive)	6 (25.0%)	36 (27.7%)	0.870 (0.283 - 2.575)	0.295 (0.027 - 3.192)
Cirrhosis (yes)	11 (45.8%)	62 (47.7%)	0.928 (0.356 - 2.410)	1.630 (0.324 - 8.188)
Alcoholism (yes)	6 (25.0%)	37 (28.5%)	0.838 (0.272 - 2.474)	1.135 (0.212 - 6.061)
Creatinine (mg/dL)	1.0 ± 0.2	1.3 ± 1.6	0.324 (0.048 - 2.202)	0.849 (0.167 - 4.305)
				
Tumor characteristics				
Microvascular invasion (yes)	11 (45.8%)	35 (26.9%)	2.297 (0.862 - 6.110)	10.560 (1.312 - 84.994)
Edmondson's grading (> 2)	18 (75.0%)	77 (59.2%)	2.065 (0.710 - 6.271)	6.315 (0.758 - 52.618)
Encapsulation (yes)	17 (70.8%)	99 (76.2%)	0.760 (0.265 - 2.243)	1.775 (0.285 - 11.055)
Tumor number (> 2)	9 (37.5%)	21 (16.2%)	3.114 (1.088 - 8.867)^e^	2.933 (0.561 - 15.334)
Microsatellites (yes)	7 (29.2%)	20 (15.4%)	2.265 (0.740 - 6.794)	2.032 (0.185 - 22.347)
Tumor size (Diameter, cm)	8.0 ± 5.2	6.7 ± 4.6	1.057 (0.970 - 1.152)	0.881 (0.700 - 1.110)
Portal Vein Thrombosis (yes)	3 (12.5%)	10 (7.7%)	1.741 (0.341 - 7.616)	0.893 (0.094 - 8.487)
Alpha-fetoprotein (100 ng/mL)	70.3 ± 394.6	31.3 ± 55.6	1.000 (0.997 - 1.002)	0.996 (0.988 - 1.003)
				
Liver Function				
Albumin (g/dL)	3.7 ± 0.6	3.8 ± 0.7	0.881 (0.446 - 1.742)	1.493 (0.409 - 5.456)
Bilirubin (mg/dL)	1.2 ± 0.9	1.3 ± 1.8	0.950 (0.688 - 1.310)	1.032 (0.588 - 1.812)
Prothrombin time (sec)	12.2 ± 1.2	12.5 ± 1.6	0.876 (0.623 - 1.232)	0.905 (0.563 - 1.456)
Ascites (yes)	2 (8.3%)	12 (9.2%)	0.894 (0.129 - 4.693)	1.140 (0.103 - 12.576)
				
Liver necroinflammation				
AST (U/L)	117.3 ± 202.2	91.1 ± 98.3	1.002 (0.998 - 1.005)	1.009 (0.998 - 1.019)
ALT (U/L)	72.3 ± 116.9	75.6 ± 91.9	1.000 (0.995 - 1.005)	0.997 (0.987 - 1.007)

^a^Unadjusted odds ratios were calculated using regression analysis for parametric data and chi-square test for categorical data.

^b^Odds ratios adjusted for all other variables in this table; ^c^P = 0.025; ^d^P = 0.023; ^e^P = 0.032.

### Western blot analysis of p53 expression in HCC

**Figure 4:**
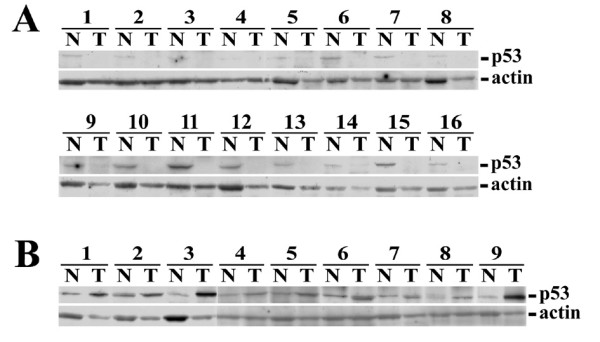
**Western blot analysis of p53 in paired cancerous (T) and adjacent non-cancerous (N) liver tissues**. (A) p53 was either undetectable or barely detected (patient-9 and 14) in the cancerous tissues. (B) p53 was clearly detectable in the cancerous liver tissues.

The cancerous and non-cancerous liver tissues were also subjected for Western blot analysis to detect p53 expression (Figure 4). Two major patterns were observed. In 129 patients, a small amount of p53 was detected in non-cancerous parts of liver tissues, whereas none (or only a trace amount) was detected in the cancerous parts of liver tissues (Figure 4A). In the remaining 25 patients, the amount of p53 in the cancerous part was greater or equal to that in the non-cancerous part (Figure 4B). Univariate analysis showed that only male gender and the presence of HBsAg were significantly correlated with p53 over-expression in HCC (Table 3). The unadjusted OR (95% CI) was 4.802 (1.019 - 31.022) and 4.203 (1.110 - 18.673), respectively (P = 0.046 and 0.031). However, in multivariate logistic regression model, these two factors were not appeared to be independent factors. CD133 expression was not associated with p53 expression in HCC.

**Table 3:** Multivariate logistic regression analysis of clinical parameters associated with p53 over-expression in HCC.

Clinicopathological parameters	p53 over-expression in HCC		Unadjusted OR **(95% CI)**^**a**^	Adjusted OR **(95% CI)**^**b**^
			
	Positive (n = 25)	Negative (n = 129)		
Age (years)	52.5 ± 14.8	56.8 ± 15.0	0.985 (0.958 - 1.013)	0.995 (0.942 - 1.050)
Sex (male)	23 (92.0%)	91 (70.5%)	4.802 (1.019 - 31.022)^c^	10.217 (0.861 - 121.307)
HBsAg (positive)	22 (88.0%)	82 (63.6%)	4.203 (1.110 - 18.673)^d^	1.490 (0.130 - 17.10)
Anti-HCV (positive)	4 (16.0%)	38 (29.5%)	0.456 (0.123 - 1.537)	0.791 (0.074 - 8.490)
Cirrhosis (yes)	14 (56.0%)	59 (45.7%)	1.510 (0.590 - 3.893)	1.639 (0.396 - 6.783)
Alcoholism (yes)	5 (20.0%)	38 (29.5%)	0.599 (0.182 - 1.857)	0.269 (0.052 - 1.390)
Creatinine (mg/dL)	1.0 ± 0.2	1.2 ± 1.0	0.614 (0.193 - 1.954)	0.106 (0.003 - 4.139)
				
Tumor characteristics				
Microvascular invasion (yes)	10 (40.0%)	36 (27.9%)	1.722 (0.647 - 4.547)	1.658 (0.249 - 11.043)
Edmondson's grading (> 2)	15 (60.0%)	93 (72.1%)	0.581 (0.220 - 1.545)	1.800 (0.404 - 8.019)
Encapsulation (yes)	18 (72.0%)	98 (76.0%)	0.813 (0.286 - 2.381)	0.600 (0.097 - 3.691)
Tumor number (> 2)	4 (16.0%)	26 (20.2%)	0.755 (0.200 - 2.609)	0.566 (0.080 - 4.008)
Microsatellites (yes)	3 (12.0%)	24 (18.6%)	0.597 (0.130 - 2.349)	2.520 (0.213 - 29.798)
Tumor size (Diameter, cm)	9.3 ± 9.5	6.9 ± 4.7	1.008 (0.920 - 1.104)	0.781 (0.590 - 1.034)
Portal Vein Thrombosis (yes)	6 (24.0%)	53 (41.1%)	0.453 (0.150 - 1.308)	4.645 (0.368 - 58.611)
Alpha-fetoprotein (100 ng/mL)	10.2 ± 27.5	62.0 ± 323.9	0.995 (0.982 - 1.008)	0.991 (0.967 - 1.016)
				
Liver Function				
Albumin (g/dL)	3.7 ± 0.7	3.8 ± 0.7	0.860 (0.436 - 1.699)	2.560 (0.644 - 10.184)
Bilirubin (mg/dL)	1.2 ± 0.8	1.3 ± 1.8	0.950 (0.688 - 1.310)	0.591 (0.273 - 1.283)
Prothrombin time (sec)	12.5 ± 1.9	12.4 ± 1.5	1.049 (0.798 - 1.379)	1.146 (0.752 - 1.745)
Ascites (yes)	3 (12.0%)	11 (8.5%)	1.463 (0.296 - 6.358)	4.545 (0.613 - 33.665)
				
Liver necroinflammation				
AST (U/L)	132.0 ± 210.7	88.7 ± 89.0	1.002 (0.999 - 1.006)	1.017 (1.005 - 1.030)
ALT (U/L)	90.4 ± 127.8	74.1 ± 91.0	1.001 (0.996 - 1.005)	0.985 (0.972 - 0.999)
CD133 expression	2 (8.0%)	22 (17.1%)	0.423 (0.064 - 2.064)	0.537 (0.055 - 5.202)

^a^Unadjusted odds ratios were calculated using regression analysis for parametric data and chi-square test for categorical data.

^b^Odds ratios adjusted for all other variables in this table;.

^c^P = 0.046; ^d^P = 0.031

### CD133 and p53 independently predict disease-free survival in HCC patients

**Figure 5:**
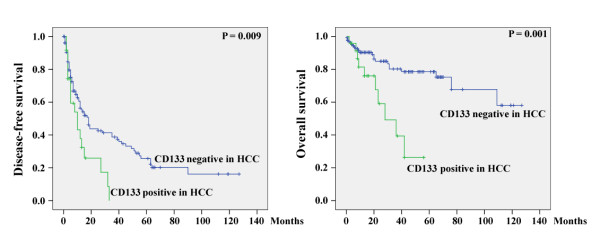
**Disease-free (left panel) and overall (right panel) survivals in patients with or without CD133 expression in HCC**.

Survival analysis indicated that patients with CD133 expression in HCC were significantly associated with poor disease-free survival (Figure 5, left). The mean (95% CI) disease-free survival time was 13.2 (8.2 - 18.3) and 40.0 (30.5 - 49.6) months respectively for patients with and without CD133 expression in HCC (P = 0.009). Additionally, CD133 expression also significantly associated with poor overall survival (Figure 5, right). The mean (95% CI) overall survival time was 32.2 (23.0 - 41.4) and 95.7 (83.7 - 107.8) months respectively for patients with and without CD133 expression in HCC (P = 0.001).

**Figure 6:**
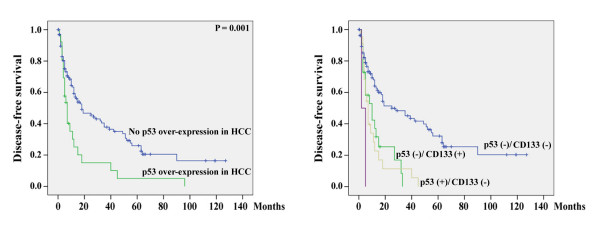
**Prediction of disease-free survival using p53 over-expression alone (left panel) or using both p53 and CD133 expression (right panel) in HCC**.

Over-expression of p53 in HCC was also significantly associated with poor disease-free survival (Figure 6). The mean (95% CI) disease-free survival time was 15.0 (5.6 - 24.5) and 40.9 (31.3 - 50.5) months respectively for patients with and without p53 over-expression in HCC (P = 0.001). However, p53 expression was not associated with overall survival. The mean (95% CI) overall survival time was 81.9 (56.8 - 106.9) and 87.6 (75.0 - 100.3) months respectively for patients with and without p53 over-expression in HCC (P = 0.606).

When the two factors were combined. It was found that patients negative for both p53 and cd133 expression in HCC have significantly better disease free-survival than those with only positive p53 expression (P < 0.001), only positive CD133 expression (P = 0.001), and both p53 and CD133 expression (P = 0.006). Although the case number in the p53(+)/CD133(+) group was small, the survival difference between this group and the p53(-)/CD133(-) group was large. As a result, the P value was significant (Figure 6, right). The mean (95% CI) overall survival time for these four groups of patients was 47.1 (35.8 - 58.4), 11.3 (5.8 - 16.8), 13.0 (8.0 - 18.0), and 3.5 (0.6 - 6.4) months, respectively.

In Cox proportional hazard analysis using only p53 and CD133 expression as covariates, it was found that the two factors were independent factors for disease-free survival. The hazard ratios (95% CI) were 1.697 (1.318 - 2.185) and 2.559 (1.519 - 4.313), respectively (P < 0.001 for both). In Cox proportional hazard analysis using all clinicopathological parameters, p53 expression, and CD133 expression as covariates, it was found that only p53 expression, tumor number, and AST remained to be significant predictors for disease-free survival. The hazard ratios (95% CI) were 1.827 (1.288 - 2.591), 1.754 (1.244 - 2.473), and 1.004 (1.001 - 1.008), respectively (P = 0.001, 0.001, and 0.008).

## Discussion

In recent years, several putative tissue-restricted stem cell markers were identified in their tumor counterparts [18,30,31]. For example, CD34^+^CD38^- ^cells in leukemia, CD44^+^CD24^low/lin- ^cells in breast cancer and CD133^+ ^cells in brain and prostate tumors [17,18,22-29]. A minor subpopulation of cancer cells possessing these markers was found to exert high tumorigenicity in mice xenograft assay [28]. In human liver, when massive necrosis and rigorous regeneration occurred, CD133 could co-expressed with c-Kit on the surface of putative hepatic progenitor cells [40]. Several studies subsequently discovered that a small population of CD133-positive cells was present in hepatoblastoma as well as HCC, raising the possibility that liver cancer was of stem cell origin [34-36].

HCC expressed some de-differentiation markers, which were also present in fetal liver, such as AFP and CK19. Expression of these markers in HCC was associated with higher tumor grade and shorter survival. Similarly, CD133 expression was also associated with poorer prognosis [35,36]. At present, it is unclear whether CD133 was a de-differentiation marker, which was expressed during the process of oncogenic transformation from mature hepatocytes to poorly differentiated cancer cells or alternatively, CD133-positive HCCs represent a subgroup of liver cancers which were of stem cell origin.

In Southeast Asia including Taiwan, a great majority of HCC patients were causatively associated with hepatitis B virus infection [2]. Chronic viral hepatitis leads to perpetuated necroinflammation of the liver and thus continuous regeneration of hepatocytes. Consequently, liver cirrhosis occurred and HCC emerged. Presumably, liver stem cells played a pivotal role in hepatocyte regeneration and were prone to accumulating mutations on the target genes, which resulted in malignant transformation. To the contrary, in the present data, CD133 expression was negatively associated with the presence of serum HBsAg in both univariate and multivariate analysis. In contrast, p53 expression was positively associated with the presence of HBsAg in univariate analysis. Therefore, it was revealed for the first time that CD133 expression occurred more frequently in HCCs unrelated to HBV infection. The present data argued against a cancer-stem-cells origin of HBV-related HCC and the CD133-positive HCCs appeared to be a distinct subset of liver cancer not originated from chronic HBV infection.

One of the most frequently reported genetic alterations in human cancers was p53 gene mutation. Wild-type p53 is believed to be a tumor suppressor, which inhibits tumor growth when expressed. Its mutated forms however, exert a dominant negative function and acts like an oncogene. As a result, cancers expressing high level of mutated p53 tend to be more aggressive and associated with poor prognosis [37,38]. Although we did not sequence the p53 gene in this study, a previous report indicated that p53 mutations in HCC were commonly seen in Taiwanese patients [41]. Consistent with previous studies, the present results also indicated that p53 over-expression was associated with shorter disease-free survival. Importantly, in Cox proportional hazard model including all clinicopathological covariates for analysis, p53 was one of the independent predictors. In contrast, CD133 was not a significant predictor in this model, suggesting that other clinicopathological predictors were associated with CD133 expression. Finally, Cox proportional hazard model clearly indicated that CD133 and p53 were two independent predictors for disease-free survival in HCC. This result demonstrated for the first time that oncogenesis of the putative stem-cell-originated HCCs likely involved pathways independent of p53 mutations.

## Conclusion

Our present data indicated that in an area endemic for HBV infection, HCCs positive for either p53 or CD133 expression were detected in a small but substantial proportion of patients (16.2% and 15.6%). These two markers independently predicted shorter disease-free survival, suggesting that growth of CD133-positive HCC involved a pathway independent of p53 mutation. Furthermore, CD133 expression was negatively associated with the presence of serum HBsAg in our patients, arguing against a stem cell origin of HBV-related HCC. The present results implicated that CD133-positive HCC was a subset of liver cancer not originated from chronic HBV infection but originated from putative "cancer stem cells".

## Competing interests

The authors declare that they have no competing interests.

## Authors' contributions

C-T Y, C-K C and C-Y L designed the study and analyzed the data. C-T Y and C-J K involved in drafting the manuscript and revising it critically for important intellectual content. M-W L performed the experiments and interpreted the Western blot data. T-C C reviewed and interpreted the pathological data. T-S Y and W-C L collected liver samples and analyzed clinical data. All authors have read and approved the final manuscript.

## Pre-publication history

The pre-publication history for this paper can be accessed here:

http://www.biomedcentral.com/1471-2407/9/324/prepub
